# 795. A Bayesian Spatial-temporal Modelling Approach for Prevalence Estimation of a VRE Outbreak in a Tertiary Care Hospital

**DOI:** 10.1093/ofid/ofab466.991

**Published:** 2021-12-04

**Authors:** Andrew Atkinson, Benjamin Ellenberger, Olga Endrich, Tanja Kaspar, Maria Hardegger, Walter Steiger, Vanja Piezzi, Alexandra Leichtle, Jonas Marschall

**Affiliations:** 1 Bern University Hospital, Bern, Switzerland; 2 University of Bern, Bern, Switzerland

## Abstract

**Background:**

There was a nosocomial outbreak of vancomycin-resistant enterococci (VRE) in our hospital group from 2018-19. The goals of the study were to describe the prevalence trajectory and explore risk factors associated with putative room colonization during the outbreak.

**Methods:**

We performed a room centric analysis of 12 floors (floors F to R, 264 rooms) of the main bed tower of the hospital, including data on 37’458 patients (23’050 person weeks) over the 104 week period. Patients were assumed to be colonized in the week prior to their first positive test, and thereafter throughout the remainder of their stay until discharge. Poisson Bayesian Hierarchical models were fitted to estimate prevalence per room, including both spatial (conditional autoregressive) and temporal (random walk) random effects terms. Model M1 estimated prevalence for each floor and then used meta-analysis to combine the estimates, whereas model M2 estimated prevalence for “all-floors” simultaneously.

**Results:**

The oncology department, where the outbreak was thought to have started, experienced slightly higher prevalence (floors O and R; adjusted incidence rate ratio (aIRR) 4.8 [2.6, 8.9], p< 0.001; reference is general medicine; see Figure Panel A), as did both the cardiac surgery (floors G, N, O; aIRR 3.8 [2.0, 7.3], p< 0.001) and abdominal surgery departments (floors H and Q; 3.7 [1.8, 7.6], p< 0.001). There was no discernible difference in prevalence between floors with single and multiple department occupancy. Furthermore, departments spread across multiple floors had similar prevalence on all constituent floors – perhaps indicating transmission by people or devices moving between floors.

The “single floor meta-analysis” model (M1) more closely followed the estimated trajectory for the crude prevalence, whereas the “all floors” model (M2) dampened the amplitude of the peaks somewhat, but better estimated periods of low prevalence (Figure Panel B).

Figure: Estimates from the Bayesian Hierarchical Models

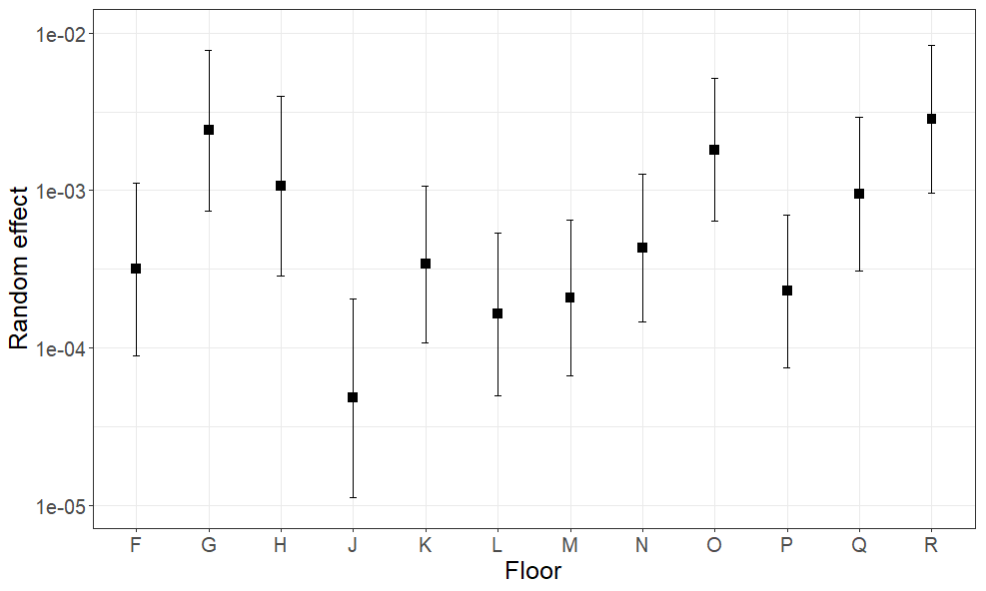

Panel A. Random effect prevalence estimates for each floor (from model M2). Panel B. Crude prevalence (black) and estimates from the “single floor meta-analysis” approach (M1, dashed red) with 95% credible intervals shaded (shaded red), and “all-floors” model (M2, blue ).

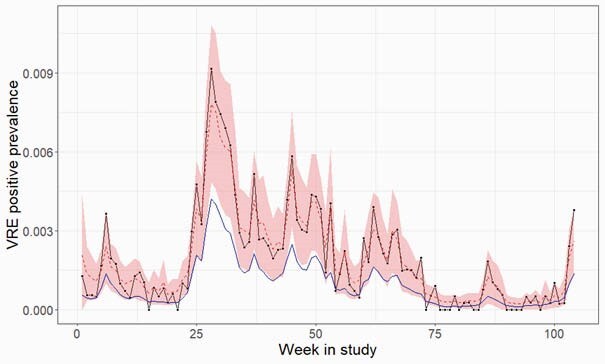

**Conclusion:**

We applied a room centric approach that took into account spatial and temporal dependencies apparent in the nosocomial VRE outbreak. Despite additional complexity, Bayesian Hierarchical Models provide a more flexible platform for studying transmission dynamics and performing hypothesis testing, compared to more traditional methods.

**Disclosures:**

**All Authors**: No reported disclosures

